# A Spectrum Correction Algorithm Based on Beat Signal of FMCW Laser Ranging System

**DOI:** 10.3390/s21155057

**Published:** 2021-07-26

**Authors:** Yi Hao, Ping Song, Xuanquan Wang, Zhikang Pan

**Affiliations:** 1 Key Laboratory of Biomimetic Robots and Systems (Ministry of Education), Beijing Institute of Technology, Beijing 100081, China; 3120190156@bit.edu.cn (Y.H.); 3120185108@bit.edu.cn (X.W.); 2 Instrument of Science and Technology, Beijing Information Science and Technology University, Beijing 100192, China; panzhikang@bistu.edu.cn

**Keywords:** FMCW laser ranging, spectrum correction, white Gaussian noise, spectrum leakage, picket fence effect, signal processing

## Abstract

The accuracy of target distance obtained by a frequency modulated continuous wave (FMCW) laser ranging system is often affected by factors such as white Gaussian noise (WGN), spectrum leakage, and the picket fence effect. There are some traditional spectrum correction algorithms to solve the problem above, but the results are unsatisfactory. In this article, a decomposition filtering-based dual-window correction (DFBDWC) algorithm is proposed to alleviate the problem caused by these factors. This algorithm reduces the influence of these factors by utilizing a decomposition filtering, dual-window in time domain and two phase values of spectral peak in the frequency domain, respectively. With the comparison of DFBDWC and these traditional algorithms in simulation and experiment on a built platform, the results show a superior performance of DFBDWC based on this platform. The maximum absolute error of target distance calculated by this algorithm is reduced from 0.7937 m of discrete Fourier transform (DFT) algorithm to 0.0407 m, which is the best among all mentioned spectrum correction algorithms. A high performance FMCW laser ranging system can be realized with the proposed algorithm, which has attractive potential in a wide scope of applications.

## 1. Introduction

The frequency modulated continuous wave (FMCW) laser ranging system is a noncontact detecting and distance measurement system, which has a large detection range and high measurement accuracy and has been widely used in high precision ranging. The system utilizes the corresponding relationship between frequency and distance, which means that the accuracy of distance value relies on the frequency resolution of beat signal obtained by a series of processing with the emitted signal and echo signal. Therefore, the key of ranging lies in the frequency calculation of beat signal [1].

The frequency value of the beat signal can be computed by discrete Fourier transform (DFT) after the signal is sampled and digitized. Ideally, the frequency resolution of it is closely related to the number of sampling points. Too few sampling points will decrease the frequency resolution and lead to the picket fence effect, which affects ranging accuracy, while too many will increase the computing time and the complexity of signal processing. An optimization method is to add points whose value are zero after the sampled beat signal [2]. The accuracy of this method for calculating the frequency completely depends on the number of added points. However, this operation is equivalent to utilizing a rectangular window function on the beat signal in the time domain. This not only cannot change the width of the main lobe in the spectrum but also causes spectrum leakage to a certain extent. Consequently, spectrum correction algorithms to improve the accuracy and resolution of the beat signal frequency have become more significant.

The ratio algorithm is the interpolation-based correction method [3,4,5]. Agrez et al. [6] and Belega et al. [7,8,9] have conducted a further studies on it and proposed new methods based on it to reduce the influence of spectrum leakage on the accuracy of correction. However, the above methods are all at the expense of noise adaptability. The phase difference (PD) algorithm originally comes from a phase interpolation estimator of a single tone frequency in noise proposed by McMahon et al. [10]. Zhu et al. [11] and Kang et al. [12] have performed further research on it, which indicates that the PD algorithm provides superior accuracy in frequency estimates compared with the ratio algorithm and has good adaptability. Luo et al. [13] proposed a new PD method based on asymmetric windows, which can be used to correct the errors of frequency. The main advantages of this algorithm are its characteristics of simple application and strong anti-noise performance, but its reduction of spectrum leakage is unsatisfactory.

The concept of energy centrobaric correction (ECC) algorithm [14] is originally proposed by Offelli et al. [15]. Many researchers have investigated the interferences from spectral components, wideband noise, and other precision factors related to the estimated parameters [16,17]. This algorithm has fast speed and great accuracy of frequency calculation, so it has been applied to engineering after improvement [18]. However, the correction accuracy is too dependent on the symmetric window function and is easily affected by white Gaussian noise (WGN). The Chirp z-transform (CZT) algorithm is a z-transformation method [19]. Because of the low complexity of calculation and the high correction accuracy, there are a lot of CZT-based related applications [20,21,22,23,24,25]. Because this algorithm still analyzes the truncated signal, it only reduces the influence of the picket fence effect on the local spectrum, whereas it does not significantly solve the problem caused by spectrum leakage. The Zoom fast Fourier transform (ZFFT) algorithm achieves spectrum correction by reducing the sampling rate of the signal. It blends complex down-conversion, low-pass filtering, and sample-rate change by way of decimation, thereby improving the frequency resolution [26]. Al-Qudsi et al. [27] presented an implementation method of the ZFFT approach to estimate the spectral peak in the FMCW radar, utilizing a field programmable gate array (FPGA). This algorithm can decrease the complexity of calculations and alleviate the influence of picket fence effect. However, it is severely affected by spectrum leakage and WGN.

In view of the unsatisfactory accuracy and resolution of beat signal frequency affected by WGN, spectrum leakage, and picket fence effect, which cannot be solved by the traditional algorithms above, we propose a new spectrum correction algorithm called decomposition filtering-based dual-window correction (DFBDWC). The main contributions are as follows:

(1) This algorithm reduces the influence of WGN, affecting the correction accuracy. In the decomposition and filter part, the beat signal is divided into several components, and each component has its characteristics in the frequency domain. Among them, the first few components possess the widest frequency coverage, and there are no obvious peaks in their power spectrum. The sum can be used as the input of the filter, and the WGN in the beat signal will be mostly removed with the weight parameter.
(2) This algorithm minimizes the impact of spectrum leakage effectively. The Hann window has a narrow main lobe, low side lobe, and fast attenuation speed from the main lobe to the first side lobe. Using two Hann windows in the correction part can concentrate more energy of the signal, thereby making the spectral peak of the desired frequency more obvious.
(3) This algorithm diminishes the picket fence effect that may decrease the frequency resolution of the beat signal. We utilize phase values and the delay value of two signals in the frequency domain after DFT processing. The phase values correspond to the spectral peaks that are at the same position in these signals. Therefore, the calculation error caused by broad adjacent spectral lines near the peak in only one used signal is avoided, and an accurate frequency value of the beat signal is obtained.
(4) This algorithm is different from the traditional spectrum correction algorithm, which can reduce the influence caused by WGN, spectrum leakage, and the picket fence effect at the same time, so that the frequency value obtained by this algorithm is more accurate and the distance ranged by this system is more precise.

This article is organized as follows. In Section 2, the principle of the FMCW laser ranging system is firstly briefly introduced, and we explain the DFBDWC algorithm in detail. In Section 3, we built an experimental platform based on the principle of the FMCW laser-ranging system. The results are obtained via simulation and experiment on this platform. Afterwards, the discussion that evaluates the spectrum correction performance of this algorithm by comparing it with these traditional algorithms is conducted. Finally, Section 4 concludes the article.

## 2. Methods

**Figure 1:**
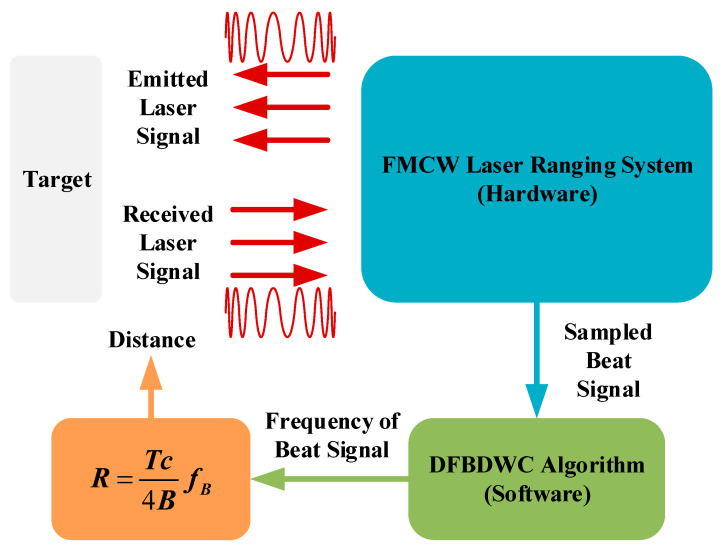
Schematic diagram of distance obtained by the FMCW laser ranging system and DFBDWC algorithm.

Using the method shown in Figure 1, we can obtain the high-precision distance value of the target.

The FMCW laser-ranging system emits a modulated laser signal that is reflected by the target and received by the system. After the processing of the received laser signal, the system will output the sampled beat signal. In the software part, we can calculate the precise frequency value of the sampled beat signal with the DFBDWC algorithm and obtain the distance value of this target by taking the frequency value into the equation. In this section, we will introduce the principle of the FMCW laser ranging system and the DFBDWC algorithm, respectively, in detail.

### 2.1. FMCW Laser Ranging System

**Figure 2:**
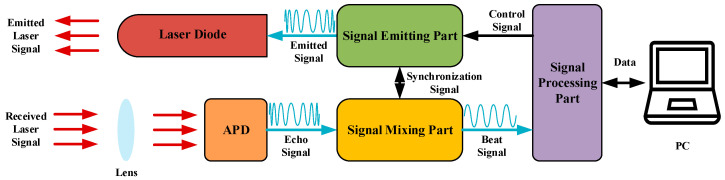
Scheme of FMCW laser ranging system.

The FMCW laser ranging system can be mainly divided into seven parts. The schematic diagram of it is as shown in Figure 2. The signal processing part controls the signal emitting part to generate the FMCW emitted signal, and it drives the laser diode to emit a linear beam, which is the emitted laser signal. The avalanche photo diode (APD) receives the laser signal that is focused by the lens and outputs the echo signal, which is a FMCW signal with a certain delay of emitted signal. The echo signal and the local oscillator signal synchronized by the signal emitting part are mixed in the signal mixing part, and with a series of processing, the beat signal is obtained. In the signal processing part, the beat signal is digitized and transformed into data, which are stored and sent to the PC. Finally, the beat signal is analyzed and processed by the algorithm in the PC, and the distance is computed.

**Figure 3:**
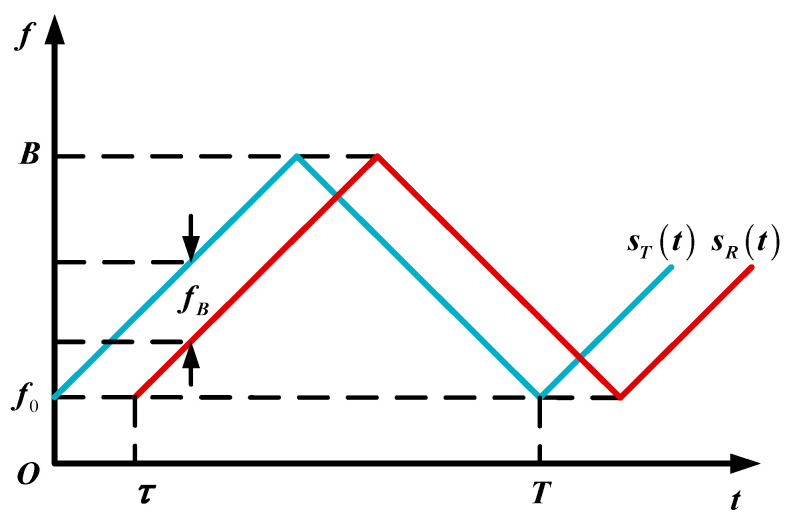
Type of FMCW modulation. The blue and red line represent the emitted signal $s_{T}\left(t\right)$ and echo signal $s_{R}\left(t\right)$, respectively.

In this FMCW laser ranging system, the frequency of the emitted signal is modulated by a triangle wave, which is as shown in Figure 3. Because of the static ranging target, the effect of the Doppler shift does not have to be considered. Then, the emitted signal $s_{T}\left(t\right)$ is expressed by

(1) $$s_{T}\left(t\right)=A_{0}\cos\left(2\pi f_{0}t+\pi kt^{2}+\varphi_{0}\right),$$

where $A_{0}$ is the amplitude of emitted signal, $f_{0}$ is the initial frequency, $\varphi_{0}$ is the initial phase, k=2B/T is the modulation slope, B is the modulation bandwidth, and T is the modulation period. With the delay τ=2R/c, the echo signal $s_{R}\left(t\right)$ can be obtained by

(2) $$s_{R}\left(t\right)=\eta A_{0}\cos\left(2\pi f_{0}\left(t-\tau \right)+\pi k{\left(t-\tau \right)}^{2}+\varphi_{0}\right),$$

where η is the amplitude decay rate of echo signal, R is the distance, and c is the speed of light. With the mixing of the emitted and echo signal, the beat signal $s_{B}\left(t\right)$ can be calculated by

(3) $$s_{B}\left(t\right)=\frac{\eta A_{0}{}^{2}}{2}\cos\left(2\pi f_{0}\tau +2\pi k\tau t-\pi k\tau^{2}\right).$$

Obviously, the frequency of the beat signal $f_{B}=k\tau$. Thus, the relationship between R and $f_{B}$ is

(4) $$R=\frac{Tc}{4B}f_{B}.$$

As shown in the above equation, the factors affecting the accuracy of FMCW laser ranging system are T, B, and $f_{B}$. Because T and B are the inherent parameters of this system, and they have already reached the limit of system performance, they do not have a decisive impact on the ranging accuracy. Improving the frequency resolution of the beat signal and obtaining the accurate $f_{B}$ become the most significant work of this system.

### 2.2. DFBDWC Algorithm

**Figure 4:**
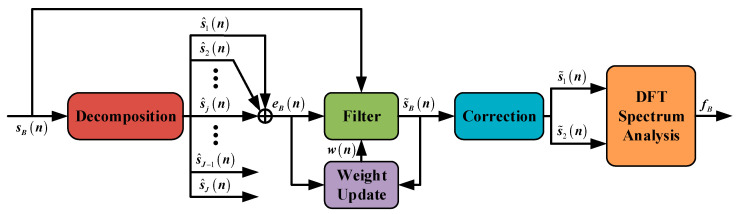
Process of DFBDWC algorithm. The input and output of this algorithm are the beat signal and frequency value of the beat signal, respectively.

This section depicts a new spectrum correction algorithm that is different from the six traditional algorithms introduced in Section 1. The key step of it, whose process chart is as shown in Figure 4, is as follows: Improving the signal to noise ratio (SNR) of $s_{B}\left(t\right)$ in decomposition and filter, extracting two sub-signals with a dual-window on each of them, and calculating accurate $f_{B}$ according to the phase values in correction and DFT spectrum analysis. All the parameters are in digital form as (n).

WGN is a kind of noise whose probability density function satisfies the statistical characteristics of normal distribution and whose power spectral density function is constant. The most noteworthy feature of it is that the signal contains all frequency components from negative infinity to positive infinity, so it can be apparently distinguished from useful signals with a spectral peak in the spectrum. Accordingly, a similar sequence of WGN can be decomposed from the beat signal.

First, decomposition is a new method based on empirical mode decomposition (EMD) [28,29,30] that is a direct extraction of the energy associated with various intrinsic time scales and the most important parameters of the system. After processing with EMD, a signal can be expressed as a sum of amplitude- and frequency-modulated functions called modes. Each mode is intrinsic and has unique characteristics in the frequency domain, which means several of them enable the estimation of WGN. However, there are some phenomena in EMD, such as oscillations with very disparate scales in one mode or oscillations with similar scales in different modes. These phenomena will cause a problem called “mode mixing”, and some decomposed modes, strictly speaking, will not be a single component signal, so it is not conducive to estimate the noise component accurately.

In order to alleviate “mode mixing”, we take advantage of the dyadic filter bank behavior of EMD and the addition of WGN that populates the whole time–frequency space. Then, the K sub-signal of $s_{B}\left(n\right)$ can be expressed by

(5) $${\left(s_{B}\left(n\right)\right)}_{k}=s_{B}\left(n\right)+{\left(-1\right)}^{k}\cdot \beta_{1}\cdot E_{1}\left(G_{k}\left(n\right)\right),$$

where $G_{k}\left(n\right)\;\left(k=1,2,\ldots ,K-1,K\right)$ is the kth added WGN signal of zero mean unit variance, K is the number of WGN signals, $E_{j}\left(\cdot \right)\;\left(j=1,2,\ldots ,J-1,J\right)$ is the jth mode obtained by EMD, and J is the number of modes or components, $\beta_{j}\;\left(j=1,2,\ldots ,J-1,J\right)$ is the jth parameter used to adjust SNR between added WGN signals and $s_{B}\left(n\right)$. The main purpose of adding the WGN signal with known features operated by EMD is to generate new extreme points. Additionally, with the operation of plus and minus, $s_{B}\left(n\right)$ will be forced to focus on some specific values in the scale energy space.

After that, let M(·) be the operator that produces the mean envelope of each signal in parentheses, which is same as the procedure in EMD and will make use of these new extreme points in parentheses. Additionally, let A(·) be the operator that produces an average signal of all signals in parentheses. With the operation of EMD, we can obtain the first component ${\hat{s}}_{1}\left(n\right)$ of beat signal $s_{B}\left(n\right)$, which is

(6) $$\begin{array}{c} {\hat{s}}_{1}\left(n\right)=A\left(E_{1}\left({\left(s_{B}\left(n\right)\right)}_{k}\right)\right)=A\left({\left(s_{B}\left(n\right)\right)}_{k}-M\left({\left(s_{B}\left(n\right)\right)}_{k}\right)\right)\\ \;=s_{B}\left(n\right)-A\left(M\left({\left(s_{B}\left(n\right)\right)}_{k}\right)\right). \end{array}$$

Averaging is meant to better estimate the mean envelope value, which reduces “mode mixing” and produces more distinct components. Because of the concept in EMD called residue, the first residue of the beat signal can be expressed by

(7) $$R_{1}\left(n\right)=s_{B}\left(n\right)-{\hat{s}}_{1}\left(n\right)=A\left(M\left({\left(s_{B}\left(n\right)\right)}_{k}\right)\right).$$

With ${\hat{s}}_{1}\left(n\right)$ and $R_{1}\left(n\right)$, we can estimate the second residue $R_{2}\left(n\right)$ and the second component ${\hat{s}}_{2}\left(n\right)$ of beat signal, respectively, by

(8) $$R_{2}\left(n\right)=A\left(M\left(R_{1}\left(n\right)+{\left(-1\right)}^{k}\cdot \beta_{2}\cdot E_{2}\left(G_{k}\left(n\right)\right)\right)\right),$$

(9) $${\hat{s}}_{2}\left(n\right)=R_{1}\left(n\right)-R_{2}\left(n\right)=R_{1}\left(n\right)-A\left(M\left(R_{1}\left(n\right)+{\left(-1\right)}^{k}\cdot \beta_{2}\cdot E_{2}\left(G_{k}\left(n\right)\right)\right)\right).$$

Similarly, for the jth(j=3,4,…,J−1,J) residue $R_{j}\left(n\right)$ and the jth(j=3,4,…,J−1,J) component ${\hat{s}}_{j}\left(n\right)$ of the beat signal can be calculated by

(10) $$R_{j}\left(n\right)=A\left(M\left(R_{j-1}\left(n\right)+{\left(-1\right)}^{k}\cdot \beta_{j}\cdot E_{j}\left(G_{k}\left(n\right)\right)\right)\right),$$

(11) $$\begin{array}{c} {\hat{s}}_{j}\left(n\right)=R_{j-1}\left(n\right)-R_{j}\left(n\right)\\ =R_{j-1}\left(n\right)-A\left(M\left(R_{j-1}\left(n\right)+{\left(-1\right)}^{k}\cdot \beta_{j}\cdot E_{j}\left(G_{k}\left(n\right)\right)\right)\right). \end{array}$$

In this way, we not only utilize the advantages of EMD to make the frequency distribution of components more obvious but also reduce the effect of “mode mixing” so as to estimate the noise components more accurately.

Next, these J components can be distinguished according to the characteristics of each component in the frequency domain. Among them, the first to jth components possess the widest frequency coverage, and there are no obvious peaks in their power spectrum. At the same time, they are scattered in the time domain. Therefore, the sum of the first to jth components is regarded as the evaluation signal $e_{B}\left(n\right)$ of $s_{B}\left(n\right)$, which can be considered to be the estimation of WGN, and we can put it into the filter.

The filter in this algorithm has three inputs and one output [31], which is expressed by

(12) $${\tilde{s}}_{B}\left(n\right)=s_{B}\left(n\right)-w\left(n\right)e_{B}\left(n\right),$$

where w(n) is a coefficient of weight, ${\tilde{s}}_{B}\left(n\right)$ is reconstruction signal of $s_{B}\left(n\right)$. In this filter, $e_{B}\left(n\right)$ is weighted by a parameter at the same instant, so we consider that it is the possible interference signal. If it is subtracted from $s_{B}\left(n\right)$, the useful information can be saved as much as possible in ${\tilde{s}}_{B}\left(n\right)$. The weight w(n) is not a fixed parameter, which needs to be updated with the input $e_{B}\left(n\right)$ and the output ${\tilde{s}}_{B}\left(n\right)$ at the same instant; then, its value of the next instant will be obtained. In order to ensure the best result of this filter, we consult the calculation method of w(n) in [31].

However, these parameters above are described in matrices or vectors according to [31], which will lead to the great cost of increased computational complexity and some stability problems. In order to reduce the complexity of computation and the cost of calculation, we have changed the order of w(n) into 1 without affecting the performance of the filter. The expression of the weight update can be expressed by

(13) $$w\left(n\right)=w\left(n-1\right)+r\left(n\right){\tilde{s}}_{B}\left(n\right)e_{B}\left(n\right),$$

where r(n) is a relevant coefficient. According to [31], it can be obtained by

(14) $$r\left(n\right)=\frac{r\left(n-1\right)}{\lambda }\left[1-g\left(n\right)e_{B}\left(n\right)\right],$$

where λ is the forgetting factor. It is introduced to give a greater forgetting effect to ${\tilde{s}}_{B}\left(n\right)$ of the latest moment, and give less forgetting effect to ${\tilde{s}}_{B}\left(n\right)$ of the farther moment, so as to ensure that ${\tilde{s}}_{B}\left(n\right)$ in the past period is “forgotten” well, so that the filter can work in a more stable state. g(n) is a coefficient of gain, which controls the effect of the output ${\tilde{s}}_{B}\left(n\right)$. Referring to the steps in [31], g(n) is calculated by

(15) $$g\left(n\right)=\frac{r\left(n-1\right)e_{B}\left(n\right)}{\lambda +r\left(n-1\right)e_{B}{}^{2}\left(n\right)}.$$

Afterwards, we utilize correction to process ${\tilde{s}}_{B}\left(n\right)$ without the interference of WGN. Correction is the processing of ${\tilde{s}}_{B}\left(n\right)$ in the time domain [32,33], which is used to reduce the picket fence effect and spectrum leakage by spectral peaks in only two sub-signals of ${\tilde{s}}_{B}\left(n\right)$ with the time delay and dual-window of each sub-signal, respectively. With the processing of this step in the DFBDWC algorithm, the calculated frequency value will be more accurate and precise. Firstly, we extract two series of sub-signals, ${\tilde{s}}_{B}{}^{\left(1\right)}\left(n\right)$ and ${\tilde{s}}_{B}{}^{\left(2\right)}\left(n\right)$, from ${\tilde{s}}_{B}\left(n\right)$. There are L points of delay between them, which means that ${\tilde{s}}_{B}{}^{\left(2\right)}\left(n\right)$ is L points behind ${\tilde{s}}_{B}{}^{\left(1\right)}\left(n\right)$. By putting the first L points of ${\tilde{s}}_{B}{}^{\left(1\right)}\left(n\right)$ and the whole points of ${\tilde{s}}_{B}{}^{\left(2\right)}\left(n\right)$ together, we can acquire ${\tilde{s}}_{B}\left(n\right)$.

After that, the first correction signal ${\tilde{s}}_{1}\left(n\right)$ can be obtained by

(16) $${\tilde{s}}_{1}\left(n\right)=S\left({\tilde{s}}_{B}{}^{\left(1\right)}\left(n\right)\cdot N\left(W\left(n\right)*W\left(n\right)\right)\right),$$

where W(n) is a window signal, which is usually the Hann window because of its excellent performance in side lobe suppression, ∗ is the operator of convolution, and N(·) is the operator of producing normalization signal. S(·) is the operator that produces a sum signal of the signal’s front and back halves in parentheses. Figuratively speaking, there is a signal whose length is 2N in parentheses of S(·); this signal’s front half means the first to Nth points and back half means the N+1th to 2Nth points. The sum signal produced by S(·) is in the length of N, which is formed by adding the values of the first and N+1th, the second and N+2th, …, the Nth points and 2Nth points.

Similarly, the second correction signal ${\tilde{s}}_{2}\left(n\right)$ can be obtained by

(17) $${\tilde{s}}_{2}\left(n\right)=S\left({\tilde{s}}_{B}{}^{\left(2\right)}\left(n\right)\cdot N\left(W\left(n\right)*W\left(n\right)\right)\right).$$

With a dual-window in the time domain, the influence of spectrum leakage can be decreased more than the one-window and none-window, that is, the energy is more concentrated in the main lobe of these signals, which is more conducive to the subsequent operation in the frequency domain. Moreover, there cannot be more than two windows applied to these sub-signals of ${\tilde{s}}_{B}\left(n\right)$, because the mathematical model of correction processing is only in two dimensions.

Finally, in order to make signals turn from time domain into the frequency domain, we process ${\tilde{s}}_{1}\left(n\right)$ and ${\tilde{s}}_{2}\left(n\right)$ with DFT to obtain their spectrum signals ${\tilde{S}}_{1}\left(q\right)$ and ${\tilde{S}}_{2}\left(q\right)$. Before DFT, ${\tilde{s}}_{1}\left(n\right)$ and ${\tilde{s}}_{2}\left(n\right)$ can be also expressed in exponential form as

(18) $${\tilde{s}}_{1}\left(n\right)=Ae^{j\left(\omega_{B}{}^{*}n+\theta \right)},$$

(19) $${\tilde{s}}_{2}\left(n\right)=Ae^{j\left[\omega_{B}{}^{*}\left(n-L\right)+\theta \right]},$$

where A is the amplitude of ${\tilde{s}}_{B}\left(n\right)$, and θ is the initial phase of ${\tilde{s}}_{B}\left(n\right)$. $\omega_{B}{}^{*}$ is the angular frequency of ${\tilde{s}}_{B}\left(n\right)$, and it can be calculated by

(20) $$\omega_{B}{}^{*}=\frac{2\pi f_{B}}{f_{S}},$$

where $f_{S}$ is the sample rate.

After the processing of ${\tilde{s}}_{B}{}^{\left(1\right)}\left(n\right)$ and ${\tilde{s}}_{B}{}^{\left(2\right)}\left(n\right)$ with DFT, we can obtain their spectrum signals ${\tilde{S}}_{1}\left(q\right)$ and ${\tilde{S}}_{2}\left(q\right)$, respectively, by

(21) $${\tilde{S}}_{1}\left(q\right)=Ae^{j\theta }F_{g}{}^{2}\left(q\Delta \omega -\omega_{0}\right),$$

(22) $${\tilde{S}}_{2}\left(q\right)=Ae^{j\left(\theta -\omega_{B}{}^{*}L\right)}F_{g}{}^{2}\left(q\Delta \omega -\omega_{0}\right),$$

where $F_{g}$ is the amplitude spectrum of W(n), q is the serial number of spectral lines, Δω is the angular frequency between each spectral lines, and $\omega_{0}$ is the initial angular frequency.

In the amplitude spectrum of ${\tilde{S}}_{1}\left(q\right)$, the corresponding serial number of its spectral peak is $q^{*}$. According to this, we can find the phase values $\varphi_{1}\left(q^{*}\right)$ and $\varphi_{2}\left(q^{*}\right)$ in the phase spectrum of ${\tilde{S}}_{1}\left(q\right)$ and ${\tilde{S}}_{2}\left(q\right)$, respectively. These phase values are expressed by

(23) $$\varphi_{1}\left(q^{*}\right)=\theta ,$$

(24) $$\varphi_{2}\left(q^{*}\right)=\theta -\omega^{*}L.$$

With Equations (23) and (24), we can only obtain an estimation of $\omega_{B}{}^{*}$ as

(25) $$\varphi_{1}\left(q^{*}\right)-\varphi_{2}\left(q^{*}\right)={\hat{\omega }}_{B}{}^{*}L.$$

This is because $\varphi_{1}\left(q^{*}\right)-\varphi_{2}\left(q^{*}\right)$ is still different from the ideal value; a compensated value of the phase difference has to be introduced. The corresponding angular frequency at the spectral peak $q^{*}$ is $2\pi q^{*}/I$, where I is the length of W(n). After the delay of L, this angular frequency will lead to an additional phase shift of $2\pi q^{*}L/I$, which will increase with this delay. Since the position of spectral peak can be observed, $2\pi q^{*}L/I$ is considered to be the compensated value of the phase difference. Then, we will calculate $\omega_{B}{}^{*}$ by

(26) $$\varphi_{1}\left(q^{*}\right)-\varphi_{2}\left(q^{*}\right)+\frac{2\pi q^{*}L}{I}=\omega_{B}{}^{*}L.$$

Eventually, according to Equations (20) and (26), the frequency value $f_{B}$ of $s_{B}\left(n\right)$ can be calculated by

(27) $$f_{B}=\frac{f_{S}}{2\pi }\left[\frac{\varphi_{1}\left(q^{*}\right)-\varphi_{2}\left(q^{*}\right)}{L}+\frac{2\pi q^{*}}{I}\right].$$

The relationship between ${\tilde{s}}_{B}{}^{\left(1\right)}\left(n\right)$ and ${\tilde{s}}_{B}{}^{\left(2\right)}\left(n\right)$ with L delay will overcome the error caused by two wide spectral lines. With $2\pi q^{*}/I$, we can compute $f_{B}$ more precisely, and the influence of the picket fence effect will be reduced well. Therefore, the frequency resolution of $s_{B}\left(n\right)$ can be improved, and the purpose of spectrum correction may be achieved.

## 3. Results and Discussion

In this section, the performance of DFBDWC algorithm is evaluated by both a simulation and an experiment. In the simulation part, an original signal is constructed with Equation (3). Furthermore, to reach the real situation as much as possible, a WGN signal with appropriate SNR value is added to it, which is regarded as a beat signal. In the experiment part, we built an experimental platform according to the scheme shown in Figure 2, and a beat signal obtained with it is analyzed and processed by this algorithm. Table 1 shows the parameters used in the simulation and the experiment.

Among them, η and $A_{0}$ are only used in the simulation. $f_{0}$, B, and T are the key parameters of the FMCW laser ranging system, which influence the theoretical accuracy of distance resolution and are determined by the direct digital synthesizer (DDS) in the experimental platform. Moreover, $f_{S}$ influences the number of sampled points in beat signal and is determined by analog-to-digital converter (ADC) in the experimental platform. $f_{U}$ and $f_{L}$ are considered to be a band-pass filter applied to the beat signal, and they are determined by the performance of the low-pass filter (LPF) in the experimental platform and T, respectively. Because τ cannot be greater than T/2, the value of $f_{L}$ is 2/T; otherwise, it will violate the principle of the FMCW laser ranging system. According to Equation (4), the distance values that this system can obtain are from 1.5141 to 22.7115 m, which correspond to $f_{L}$ and $f_{U}$, respectively. Moreover, because the minimum sample time is T/2, the maximum range resolution of the system is 1.5141 m, which is given by DFT. In order to ensure the best working states of this platform and verify the performance of this algorithm better, the test distance values are shown in Table 2.

### 3.1. Simulation

**Figure 5:**
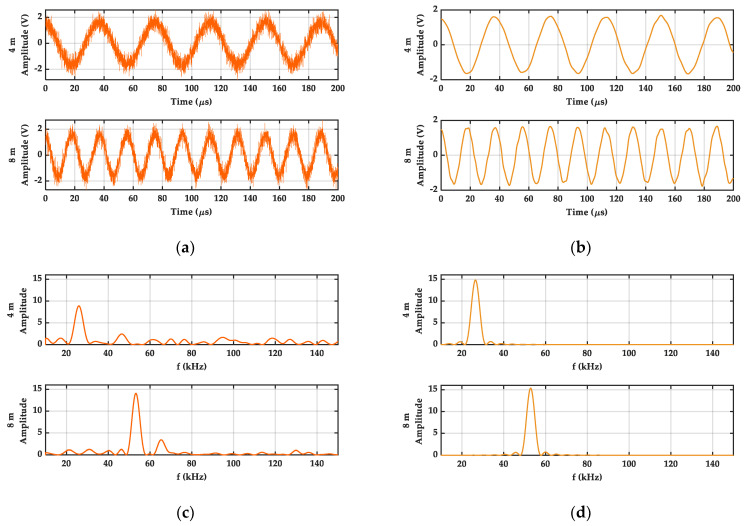
Simulation results of signals comparison at two different distances. (**a**) Beat signals $s_{B}\left(n\right)$ obtained by original signals and WGN signals with SNR of 10 dB. (**b**) Reconstruction signals ${\tilde{s}}_{B}\left(n\right)$. (**c**) The power spectrum of $s_{B}\left(n\right)$. (**d**) The power spectrum of ${\tilde{s}}_{B}\left(n\right)$.

As shown in Figure 5, it is a comparison diagram of ${\tilde{s}}_{B}\left(n\right)$ with $s_{B}\left(n\right)$ at two specific distances in both the time and frequency domain.

It can be seen that there is a great interference in $s_{B}\left(n\right)$. Compared with $s_{B}\left(n\right)$, the WGN interference in ${\tilde{s}}_{B}\left(n\right)$ is reduced well, and the wave pattern of ${\tilde{s}}_{B}\left(n\right)$ becomes smoother. Additionally, ${\tilde{s}}_{B}\left(n\right)$ also retains the information of shape, amplitude, and frequency. In their power spectrum, the amplitude of the spectral peak in ${\tilde{s}}_{B}\left(n\right)$ is greater than the amplitude of the spectral peak in $s_{B}\left(n\right)$. Moreover, the power spectrum of WGN has also been decreased. The above results indicate that the DFBDWC algorithm can suppress the interference of WGN with a large SNR value in the beat signal and retain the useful information in the signal.

In order to verify the comparison of these signals further, we apply SNR and noise power $P_{noise}$ here. They can be expressed, respectively, by

(28) $$SNR=10\mathrm{lg}\left(\frac{\sum_{n=1}^{N}x^{2}\left(n\right)}{\sum_{n=1}^{N}{\left[x\left(n\right)-s_{O}\left(n\right)\right]}^{2}}\right),$$

(29) $$P_{noise}=\frac{1}{N}\sum_{n=1}^{N}{\left[x\left(n\right)-s_{O}\left(n\right)\right]}^{2},$$

where x(n) represents $s_{B}\left(n\right)$ or ${\tilde{s}}_{B}\left(n\right)$. SNR judges the macroscopic performance of this algorithm. The larger the SNR is, and the smaller $P_{noise}$ is, the better the performance of this algorithm is, that is, the energy will be more concentrated in the position of the spectral peak and the probability of selecting a “fake” peak as the target position due to enormous WGN will be reduced better.

**Figure 6:**
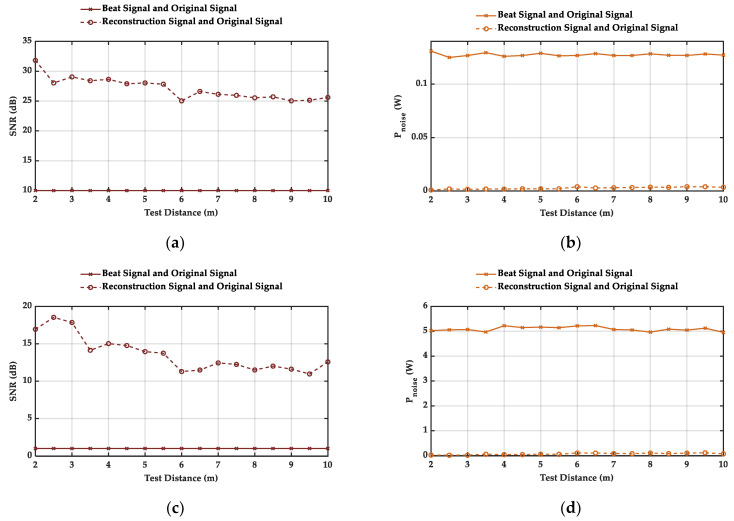
Simulation results of two parameters for evaluating signal comparison at different distances and noise powers. (**a**,**c**) are SNR, (**b**,**d**) are $P_{noise}$. Additionally, the original SNR of WGN in (**a**,**b**) is 10 dB, and the original SNR of WGN in (**c**,**d**) is 1 dB.

We calculate the value of these parameters at different distances and noise powers, and the results are as shown in Figure 6. Among the first two figures, SNR increases from 10 dB to more than 25 dB, and $P_{noise}$ reduces from 0.13 W to the order of $10^{-3}\;W$. In the last two figures, SNR increases from 1 dB to more than 11 dB, and $P_{noise}$ reduces from more than 5 W to about 0.1 W. The simulation results above indicate that this algorithm has a great suppression effect on WGN interference, and it saves useful information in ${\tilde{s}}_{B}\left(n\right)$ as much as possible. This will improve the accuracy of $f_{B}$.

Furthermore, we compare the performance of the DFBDWC algorithm with six other algorithms in terms of computing $f_{B}$, that is, the computed distances, and the original SNR of WGN in $s_{B}\left(n\right)$ is 10 dB. To show the results of the comparison better, absolute error (AE) and root mean square error (RMSE) are applied here. They can be expressed, respectively, by

(30) $$AE=\left|R_{C}\left(p\right)-R_{T}\left(p\right)\right|,$$

(31) $$RMSE=\sqrt{\frac{1}{P}\sum_{p=1}^{P}{\left[R_{C}\left(p\right)-R_{T}\left(p\right)\right]}^{2}},$$

where $R_{C}\left(p\right)$ is the computed distance of the pth test distance, $R_{T}\left(p\right)$ is the pth test distance, and P is the number of test distance. The smaller the RMSE is, the better the performance of the algorithm is.

**Figure 7:**
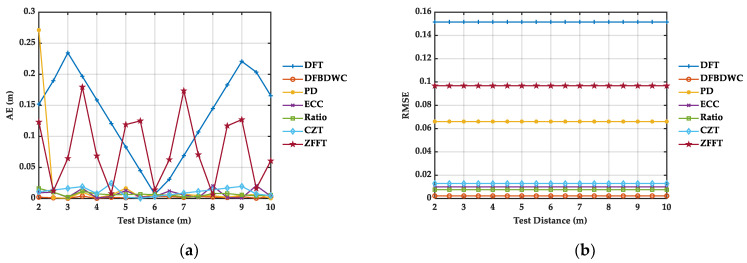
Simulation results of seven algorithms comparison at different distances. There are WGN signals with SNR of 10 dB in $s_{B}\left(n\right)$. (**a**) is AE. (**b**) is RMSE.

The computed distance and calculation results of AE and RMSE can be seen from Table 3 and Figure 7, respectively. At the first test distance, AE of the PD algorithm has the maximum value, and at the last test distance, AE of DFT algorithm has the maximum value. Among these traditional spectrum algorithm, DFT algorithm has the biggest jump of AE, while the ECC algorithm has the smallest jump of AE. The AE of the Ratio, ECC, CZT, and ZFFT algorithm are relatively stable. At each test distance, the AE of the DFBDWC algorithm basically has the minimum value. RMSE macroscopically evaluates the deviation between $R_{C}\left(p\right)$ and $R_{T}\left(p\right)$. It can be seen that the DFT algorithm has the maximum value, and the DFBDWC algorithm has the minimum value, which indicates that the DFBDWC algorithm performs the best compared with other traditional algorithms.

Additionally, in order to illustrate that this algorithm can reduce the influence of the picket fence effect and spectrum leakage further, we conduct a simulation with $s_{B}\left(n\right)$ without WGN, that is $s_{O}\left(n\right)$, at different distances, whose results of AE are as shown in Table 4. The DFBDWC algorithm still basically has the most minimum value of AE among all the spectrum correction algorithms. According to [34,35], the picket fence effect and spectrum leakage significantly decrease the precision of DFT when applying asynchronous sampling in practical applications. Additionally, there are disadvantages of each traditional spectrum correction algorithm that are described in Section 1. Therefore, the distance calculation accuracy of the DFBDWC algorithm is much better than any other six algorithms when processing with $s_{O}\left(n\right)$, which can prove our new spectrum correction algorithm not only decreases the influence of spectrum leakage, but also reduces the picket fence effect.

The simulation results demonstrate that the DFT algorithm cannot accurately obtain the distance value, since it cannot overcome the problems described in Section 1. Although the other five algorithms have improved the accuracy of $R_{C}\left(p\right)$ compared with the DFT algorithm and they have achieved a certain spectrum correction effect, they are still inferior to the performance of the DFBDWC algorithm. As a consequence, our algorithm will improve the accuracy of distance calculation.

### 3.2. Experiment

**Figure 8:**
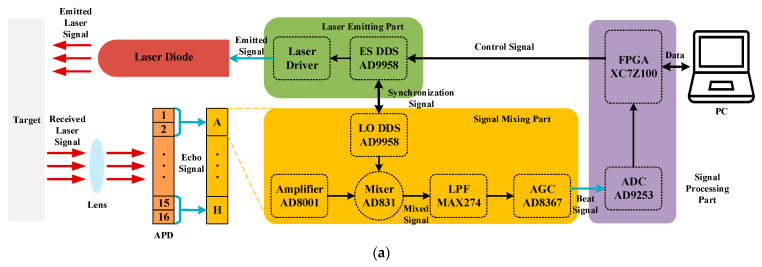

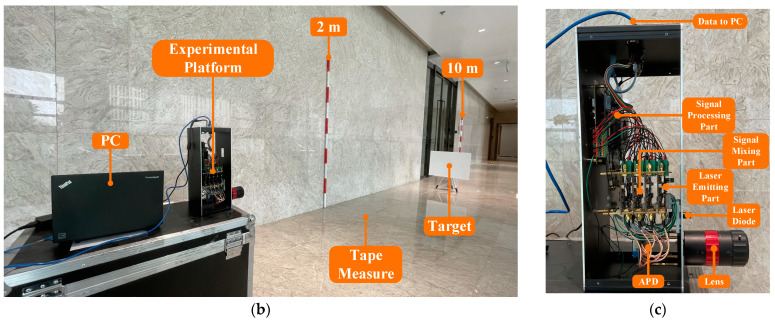
The experimental platform and scene in the experiment part. (**a**) Scheme of this experimental platform. (**b**) Experimental platform in the experimental scene. (**c**) Details of this experimental platform.

The experimental platform and scene in the experiment part are as shown in Figure 8.

The laser diode whose power is 500 mW and wavelength is 950 nm is driven by an emitted signal generated by an emitted signal (ES) DDS named AD9958. The laser beam is reflected on the target surface at $R_{T}\left(p\right)$ and focused by the lens; then, the echo signal is outputted by an APD with 16 linearly arrayed receiving units. In each signal mixing part, two series of echo signals can be processed. However, the echo signal is too weak and needs to be amplified to a certain amplitude by an amplifier named AD8001, after which a local oscillator (LO) DDS synchronized by ES DDS is mixed with it in a mixer named AD831 to form a mixed signal that contains two frequency values because of the working principle in the mixer. The large frequency value, which is an interference, needs to be filtered out by an LPF named MAX274 whose bandwidth is 150 kHz, and the small frequency value passed through an automatic gain control (AGC) named AD8367 is amplified. Then, the beat signal of appropriate amplitude can be obtained. In the signal processing part, the beat signal is sampled by an ADC named AD9253. Finally, the data are transferred to a FPGA named XC7Z100 for temporary storage and transmitted to the PC for the distance calculation using the DFBDWC algorithm. The experimental platform is placed in a corridor with a length of 15 m, and the distance between the target and APD is considered to be $R_{T}\left(p\right)$, whose value is shown in Table 2. In order to place the target in a precise position, a tape measure with centimeter accuracy is used specially, and its starting position is the surface of APD. At the same time, two benchmarks are placed at 2 and 10 m, respectively, to indicate the placement range of the target.

**Figure 9:**
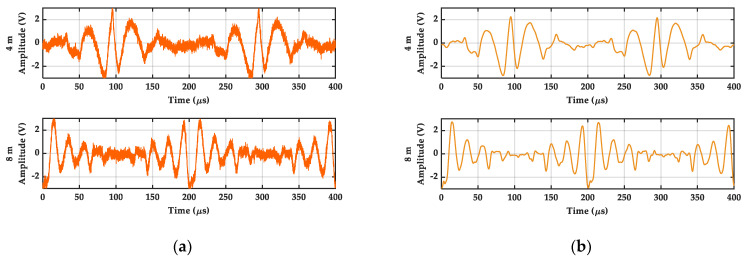
Experimental results of signals comparison at two different distances in time domain. (**a**) is beat signals $s_{B}\left(n\right)$. (**b**) is reconstruction signals ${\tilde{s}}_{B}\left(n\right)$.

Above all, we utilize only one of the units in APD to receive the laser signal from a plane target. With the analysis and processing of the DFBDWC algorithm, we obtain a comparison diagram of ${\tilde{s}}_{B}\left(n\right)$ and $s_{B}\left(n\right)$ at two specific distances in the time domain, which is as shown in Figure 9. It can be seen that they are different from ${\tilde{s}}_{B}\left(n\right)$ and $s_{B}\left(n\right)$ in the simulation part, but they still contain the frequency information of $s_{B}\left(n\right)$. Compared with $s_{B}\left(n\right)$, the WGN interference in ${\tilde{s}}_{B}\left(n\right)$ is reduced well, and the wave pattern of ${\tilde{s}}_{B}\left(n\right)$ becomes smoother. This indicates that the algorithm can suppress the interference of noise in the beat signal and retain the useful information in the signal.

**Figure 10:**
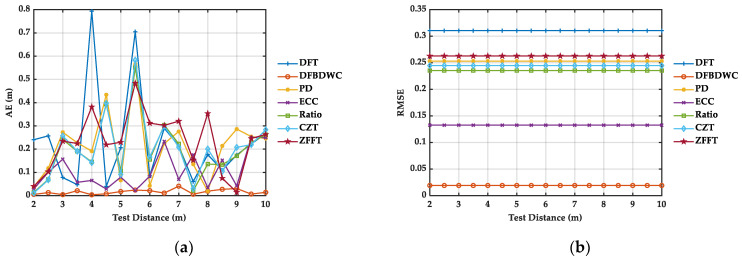
Experimental results of seven algorithms comparison at different distances. (**a**) is AE. (**b**) is RMSE.

Similarly, we compare the performance of the DFBDWC algorithm with other six algorithms in terms of computed distances in Table 5 and error analysis in Figure 10. We can note from Figure 10 that as for the other six algorithms, the AE of DFT and CZT algorithm has the maximum value, respectively, at the first and the last test distance. Overall, the DFT algorithm has the biggest jump of AE, while the ECC algorithm has the smallest jump of AE. At each test distance, the AE and RMSE of the DFBDWC algorithm basically have the minimum value. This indicates that the performance of this algorithm is the best in all these algorithms. Additionally, the maximum and minimum AE of each algorithm are listed in Table 6. The maximum AE is decreased from 0.7937 to 0.0407 m by using the DFBDWC algorithm. As expected, this algorithm overcomes the problem to a certain extent caused by spectrum leakage and the picket fence effect and improves the accuracy of distance calculation.

On the basis of experimental results above, we can conclude that because of decomposition and filter, WGN has been estimated accurately from the beat signal and reduced to a certain extent, which will improve the SNR of the beat signal. Additionally, due to the dual-window applied in correction, the energy is more concentrated and the influence of spectrum leakage has been decreased. Moreover, utilizing two main spectral lines at the peaks of these sub-signals with delay to calculate the frequency of the beat signal has alleviated the problem of poor accuracy of results caused by the picket fence effect, and it also avoid the interference that arises from using multiple spectral lines in some traditional spectrum correction algorithms. When the beat signal is not affected by WGN severely, correction will become the key step that makes the distance calculation more accurate in the DFBDWC algorithm.

In addition to the accuracy comparison of computed distance, we also calculate computation time consumed for these seven algorithms by processing the same group of sampled beat signals so as to judge the efficiency of each algorithm. Using an PC with CPU of Intel i7-7700 and RAM of 16 GB, we obtain the results that are as shown in Table 7. It can be seen that in different sample times, the computation time consumption of the ZFFT algorithm is the least, the DFBDWC algorithm is the most, and the other algorithms are almost the same. Additionally, with the increase in sample time, the computation time consumption of the DFBDWC algorithm is doubled, and there are not many rises in the other algorithms. This is because decomposition and filter in this algorithm have to process by iterative operation. The larger the sampled points of the beat signal, the more computation time consumption will be needed. In practical application, we only focus on the accuracy of the distance calculation, while we do not require any real-time computation. Therefore, we sacrifice the efficiency for the precision of our algorithm.

It can be found from the experimental results that the performance of each algorithm is consistent with simulation results. This indicates that simulation has achieved the real situation well, and the parameters for evaluating the performance of these algorithms is also reasonable. However, the values of each parameter obtained in experiment are larger than those in simulation, which is caused by errors from the experimental platform, the factors of the environment and the target placement, such as the sensitivity of APD, the response speed of the laser diode, the bandwidth of the emitted signal, the interference of ambient light, and the accuracy of distance and angle when we place the target. This can still verify that the DFBDWC algorithm reduces the influence of WGN, spectrum leakage, and the picket fence effect. Moreover, it performs the best among the existing spectrum correction algorithms, and the maximum AE of it is not more than 0.05 m.

## 4. Conclusions

In this article, we proposed a new spectrum correction algorithm named DFBDWC, and built an experimental platform based on the principle of the FMCW laser ranging system. The experimental platform outputs the data of the beat signal, which is analyzed and processed by the DFBDWC algorithm in the PC, and the target distance detected by the system is obtained. Comparing this algorithm to the traditional DFT algorithm and other spectrum correction algorithms in both the simulation and the experiment, including the PD, ECC, Ratio, CZT, and ECC algorithm, we achieve the performance evaluation of this algorithm. The results indicate that DFBDWC algorithm can reduce the influence of WGN, spectrum leakage, and the picket fence effect. Additionally, it can also improve the accuracy and frequency resolution of the beat signal. The maximum absolute error of the target distance calculated by this algorithm is reduced from 0.7937 m of the DFT algorithm to 0.0407 m, which is the best among all the spectrum correction algorithms. The most remarkable performance of our algorithm is because decomposition can estimate WGN accurately in the beat signal and the filter reduces it to a certain extent. The double Hann window applied in correction concentrates more energy in the spectrum, which minimizes the impact of spectrum leakage well. Utilizing two main spectral lines at the peaks of these sub-signals with a delay to calculate the frequency of the beat signal has alleviated the problem of poor accuracy of results caused by the picket fence effect, and it also avoids the interference that arises from using multiple spectral lines in some traditional spectrum correction algorithms. Therefore, the DFBDWC algorithm can improve the performance of the FMCW laser ranging system. In future work, it is necessary to upgrade this platform of the system, such as by choosing more sensitive APD, selecting a laser diode with a faster response speed, and increasing the bandwidth of the emitted signal, which makes it adapt to this algorithm better. In addition, we still need to optimize the structure and computational complexity of our algorithm so that the efficiency of distance calculation in engineering can be greatly raised while keeping the accuracy. Furthermore, we will carry out experiments by utilizing 16 units in APD to figure out the surface fitting uncertainty for different object shapes of this system so that it could make our algorithm and platform more valuable for 3D scanning.

## Figures and Tables

**Table 1:** The parameters used in the simulation and experiment.

Parameter	Interpretation	Value
η	The amplitude decay rate of echo signal	0.8
$A_{0}$	The amplitude of emitted signal	1
$f_{0}$	The initial frequency	1 MHz
c	The speed of light	299,792,458 m/s
B	The modulation bandwidth	99 MHz
T	The modulation period	200 μs
$f_{S}$	The sample rate	20 MHz
$f_{U}$	The upper limit frequency	0.8
$f_{L}$	The lower limit frequency	1

**Table 2:** The test distance value used in the simulation and experiment.

Lower Limit (m)	Test Distance (m)									Upper Limit (m)
1.5141	2	3	4	5	6	7	8	9	10	22.7115
	2.5	3.5	4.5	5.5	6.5	7.5	8.5	9.5		

**Table 3:** Simulation results of computed distances comparison between seven algorithms. There are WGN signals with SNR of 10 dB in $s_{B}\left(n\right)$.

Test Distance (Real Distance) (m)	Computed Distance (m)						
	DFT	PD	ECC	Ratio	CZT	ZFFT	DFBDWC
2	1.8483	2.2712	2.0092	2.0162	2.0109	2.1230	2.0021
2.5	2.3103	2.5013	2.4898	2.5102	2.5136	2.5121	2.5011
3	3.2345	2.9997	3.0018	2.9977	3.0164	2.9354	3.0004
3.5	3.6965	3.5102	3.5163	3.5118	3.5191	3.6794	3.5038
4	4.1586	3.9989	3.9992	4.0074	3.9923	4.0685	4.0003
4.5	4.6207	4.4945	4.4967	4.4939	4.5246	4.4919	4.4981
5	5.0827	4.9836	5.0128	5.0064	4.9977	4.8810	4.9988
5.5	5.5448	5.4979	5.4977	5.5069	5.5005	5.6250	5.5021
6	6.0069	5.9938	5.9968	5.9938	6.0032	6.0141	6.0028
6.5	6.4689	6.5056	6.5119	6.5053	6.5059	6.4374	6.4969
7	6.9310	6.9930	6.9942	7.0027	7.0086	6.8265	7.0017
7.5	7.3931	7.4954	7.4987	7.4968	7.5114	7.5705	7.5032
8	7.8552	7.9956	8.0202	8.0079	8.0141	7.9939	8.0022
8.5	8.3172	8.5020	8.5015	8.5079	8.5168	8.3830	8.4985
9	8.7793	8.9948	9.0008	8.9943	9.0196	9.1270	9.0030
9.5	9.7034	9.5065	9.5206	9.5043	9.4927	9.5161	9.4996
10	10.1655	9.9997	10.0038	10.0056	9.9954	9.9394	10.0027

**Table 4:** Simulation results of AE comparison between seven algorithms. There are no WGN signals in $s_{B}\left(n\right)$.

Test Distance (Real Distance) (m)	*AE* (cm)						
	DFT	PD	ECC	Ratio	CZT	ZFFT	DFBDWC
2	15.1729	27.1155	0.7593	1.6049	1.0918	12.2982	0.0064
2.5	18.9662	0.3612	0.5100	1.2172	1.3648	1.2092	0.0066
3	23.4474	0.1285	0.0044	0.3879	1.6378	6.4557	0.0118
3.5	19.6541	0.4905	0.9653	0.9247	1.9107	17.9420	0.0069
4	15.8609	0.0843	0.4251	0.7488	0.7735	6.8530	0.0112
4.5	12.0677	0.1837	0.0044	0.3630	2.4567	0.8119	0.0022
5	8.2744	0.0604	1.1222	0.6495	0.2276	11.9009	0.0109
5.5	4.4812	0.1957	0.3470	0.5298	0.0453	12.4969	0.0273
6	0.6880	0.2423	0.0084	0.3479	0.3183	1.4078	0.0087
6.5	3.1053	0.2089	1.2866	0.5007	0.5913	6.2571	0.0013
7	6.8985	0.2696	0.2793	0.4013	0.8642	17.3461	0.0036
7.5	10.6917	0.3016	0.0156	0.3363	1.1372	7.0517	0.0027
8	14.4850	0.2962	1.4637	0.4075	1.4102	0.6132	0.0093
8.5	18.2782	0.3348	0.2210	0.3157	1.2741	11.7023	0.0044
9	22.0714	0.3613	0.0266	0.3265	1.9561	12.6955	0.0084
9.5	20.3421	0.3672	1.6550	0.3435	0.7282	1.6065	0.0054
10	16.5489	0.3971	0.1714	0.2539	0.4552	6.0584	0.0024

**Table 5:** Experimental results of computed distances comparison between seven algorithms. There are WGN signals with SNR of 10 dB in $s_{B}\left(n\right)$.

Test Distance (Real Distance) (m)	Computed Distance (m)						
	DFT	PD	ECC	Ratio	CZT	ZFFT	DFBDWC
2	2.2406	2.0378	2.0288	2.0142	2.0112	2.0405	1.9947
2.5	2.2428	2.3821	2.3995	2.4305	2.4324	2.3972	2.5130
3	3.0778	3.2725	3.1569	3.2413	3.2570	3.2344	3.0044
3.5	3.4525	3.2725	3.4428	3.3091	3.3091	3.2757	3.4788
4	4.7937	4.1913	3.9345	4.1448	4.1420	4.3819	4.0037
4.5	4.5397	4.9340	4.5286	4.8935	4.8972	4.7189	4.4921
5	4.7935	4.9340	5.0797	4.8935	4.9086	4.7702	5.0177
5.5	4.7951	4.9340	5.4775	4.9486	4.9170	5.0159	5.5240
6	6.0848	6.0423	6.0834	6.1554	6.1661	6.3121	5.9786
6.5	6.7901	6.7241	6.2667	6.8037	6.7990	6.8023	6.5111
7	6.7856	6.7241	7.0700	6.7772	6.7912	6.6798	6.9593
7.5	7.5608	7.6350	7.6754	7.5213	7.5307	7.6526	7.5062
8	7.8234	7.9807	7.9655	7.8641	7.7992	7.6465	8.0192
8.5	8.3899	8.7136	8.3474	8.3670	8.3959	8.5749	8.5271
9	9.1752	8.7136	9.0420	9.1708	9.2079	9.0146	8.9694
9.5	9.7220	9.7542	9.7507	9.7275	9.7192	9.7459	9.4933
10	9.7324	9.7542	9.7507	9.7374	9.7163	9.7367	10.0143

**Table 6:** Maximum and minimum AE of each algorithm.

	DFT	PD	ECC	Ratio	CZT	ZFFT	DFBDWC
Maximum AE (m)	0.7937	0.5660	0.2507	0.5514	0.5830	0.4841	0.0407
Minimum AE (m)	0.0397	0.0193	0.0225	0.0142	0.0112	0.0146	0.0037

**Table 7:** The comparison of average computation time consuming between seven algorithms.

Sample Time (μs)	Computation Time Consuming (s)						
	DFT	PD	ECC	Ratio	CZT	ZFFT	DFBDWC
100	0.0423	0.0449	0.0458	0.0452	0.0441	0.0243	2.3891
200	0.0444	0.0501	0.0468	0.0464	0.0460	0.0259	5.6616

## Data Availability

Not applicable.
